# Diagnostic and Prognostic Protein Biomarkers of β-Cell Function in Type 2 Diabetes and Their Modulation with Glucose Normalization

**DOI:** 10.3390/metabo12030196

**Published:** 2022-02-22

**Authors:** Abu Saleh Md Moin, Thozhukat Sathyapalan, Stephen L. Atkin, Alexandra E. Butler

**Affiliations:** 1 Research Department, Royal College of Surgeons in Ireland, Adliya 15503, Bahrain; amoin@rcsi.com (A.S.M.M.); satkin@rcsi.com (S.L.A.); 2 Academic Endocrinology, Diabetes and Metabolism, Hull York Medical School, Hull YO10 5DD, UK; Thozhukat.Sathyapalan@hyms.ac.uk

**Keywords:** biomarkers, prognostic, diagnostic, type 2 diabetes, euglycemia, glucose variability

## Abstract

Development of type-2 diabetes(T2D) is preceded by β-cell dysfunction and loss. However, accurate measurement of β-cell function remains elusive. Biomarkers have been reported to predict β-cell functional decline but require validation. Therefore, we determined whether reported protein biomarkers could distinguish patients with T2D (onset < 10-years) from controls. A prospective, parallel study in T2D (*n* = 23) and controls (n = 23) was undertaken. In T2D subjects, insulin-induced blood glucose normalization from baseline 7.6 ± 0.4 mmol/L (136.8 ± 7.2 mg/dL) to 4.5 ± 0.07 mmol/L (81 ± 1.2 mg/dL) was maintained for 1-h. Controls were maintained at 4.9 ± 0.1 mmol/L (88.2 ± 1.8 mg/dL). Slow Off-rate Modified Aptamer (SOMA) -scan plasma protein measurement determined a 43-protein panel reported as diagnostic and/or prognostic for T2D. At baseline, 9 proteins were altered in T2D. Three of 13 prognostic/diagnostic proteins were lower in T2D: Adiponectin (*p* < 0.0001), Endocan (*p* < 0.05) and Mast/stem cell growth factor receptor-Kit (KIT) (*p* < 0.01). Two of 14 prognostic proteins [Cathepsin-D (*p* < 0.05) and Cadherin-E (*p* < 0.005)], and four of 16 diagnostic proteins [Kallikrein-4 (*p* = 0.001), Aminoacylase-1 (*p* = 0.001), Insulin-like growth factor-binding protein-4 (IGFBP4) (*p* < 0.05) and Reticulon-4 receptor (RTN4R) (*p* < 0.001)] were higher in T2D. Protein levels were unchanged following glucose normalization in T2D. Our results suggest that a focused biomarker panel may be useful for assessing β-cell dysfunction and may complement clinical decision-making on insulin therapy. Unchanged post-glucose normalization levels indicate these are not acute-phase proteins or affected by glucose variability.

## 1. Introduction

The chronic hyperglycemia of type 2 diabetes (T2D) results from a combination of progressive pancreatic islet β-cell loss or failure coupled with increased insulin resistance (IR) in key peripheral tissues—namely, liver, skeletal muscle and adipose tissue [1,2,3]. In the development of T2D, obesity is a well-recognized risk factor [4,5], likely due to the increased insulin resistance that obesity bestows upon the individual [6], though the deficit in β-cell mass due to apoptosis represents the core element underpinning the pathogenesis of T2D [2,7].

Functional β-cell mass denotes adequate numbers of appropriately functioning β-cells, with a deficiency in either the number or their function/identity contributing to the diminution of “functional β-cell mass” [8,9]. Although β-cell mass *per se* may not be a perfect reflection of functional β-cell mass, it does demonstrate a clear relationship with fasting blood glucose [10], and loss of identity (β-cell dedifferentiation) appears to play a minor role in the β-cell mass reduction of T2D in humans [11,12].

Clinically, however, there is no readily available, validated method to directly and accurately determine and track β-cell mass or reserve [13]. From a clinical perspective, the presence of a measure of β-cell mass would be of importance, as it may guide when insulin therapy needs to be introduced to ensure maintenance of glycemic control within the therapeutic paradigm [14].

Novel prognostic and diagnostic biomarkers have been suggested following a study of aptamer based proteomics, together with measurement of microRNA, in subjects with impaired glucose tolerance (IGT); plasma analytics were compared in the group where IGT declined over time versus an IGT group where the markers remained stable over the 3-year follow-up period [15]. The authors showed that 43 prognostic/diagnostic factors were identified that may allow monitoring of β-cell function and/or prediction of future decline. However, what is unknown is whether these biomarkers subsequently lose discrimination with the onset of T2D or may, conversely, have continued utility that may help guide clinical practice. Therefore, this study was undertaken using the same aptamer based proteomic platform of this study [15] to determine if these recognized prognostic and diagnostic markers had utility in established T2D.

## 2. Research Design and Methods

### 2.1. Study Design

This prospective case-controlled study included subjects with T2D (n = 23) and non-diabetic control (n = 23) Caucasian subjects, all aged 40–70 years. “Approval for the trial was granted by the North West-Greater Manchester East Research Ethics Committee (REC number:16/NW/0518), registered at www.clinicaltrials.gov (accessed on 10 December 2021) (NCT03102801) and conducted according to the Declaration of Helsinki. Participants provided written informed consent.

The general inclusion criteria for the T2D cohort included a duration of diabetes <10-years plus a stable dose of medication (metformin, statin and/or angiotensin converting enzyme inhibitor/angiotensin receptor blocker) maintained over the prior 3-months. Further inclusion criteria for T2D included only metformin as anti-diabetic therapy, HbA1c < 10% (86 mmol/mol) and no history of hypoglycemia/hypoglycemic unawareness during the prior 3-months. Diabetes was excluded in the control group by a normal oral glucose tolerance test (OGTT). All participants had a body mass index (BMI) of 18–49 kg/m^2^, normal renal/hepatic biochemical indices, no history of cancer, and no contraindication to insulin infusion.

A medical history, clinical examination and routine blood tests were performed; all participants had a normal electrocardiogram (ECG)” [16]. None of the patients with T2D enrolled in this study had any evidence of microvascular or macrovascular diabetes-related complications. (Table 1).

### 2.2. Hyperinsulinemic Clamp

The method for performing the insulin clamp has been previously described [16]. “Briefly, after an overnight fast, bilateral ante-cubital fossa indwelling cannulae were inserted 30 to 60 min prior to beginning the clamp (8:30 a.m.). To induce hypoglycemia, soluble intravenous insulin (Humulin S; Eli Lilly, Liverpool, UK) was given in a pump starting at a dose of 2.5 mU/kg body weight (BW)/min, with an increment of 2.5 mU/Kg BW/min every 15-min until two venous blood glucose (BG) readings measured by glucose analyser (HemoCue glucose 201+, Sweden) of 2.2 mmol/L (<40 mg/dL) or a single reading of 2.0 mmol/L (36 mg/dL) was achieved. The blood sample schedule was subsequently timed with respect to the point of hypoglycemia. Immediately following the point of hypoglycemia, intravenous glucose was given (150 mL of 10% dextrose) and repeat BG measurements performed after 5 min if BG was <4.0 mmol/L. All patients achieved a BG of 2.0 mmol/L (36 mg/dL) at the point of hypoglycemia”.

### 2.3. Blood Sample Preparation and Biochemical Marker Analyses

“Venous blood samples were collected during the screening visit. Blood samples were separated immediately by centrifugation at 3500× *g* for 15 min at 4 °C; aliquots were stored at −80 °C, within 30-min of collection, until batch analysis. Serum insulin was assayed using a competitive chemiluminescent immunoassay on the manufacturer’s DPC Immulite 2000 analyser (Euro/DPC, Llanberies, UK), with a coefficient of variation (CV) of 6 and no stated cross-reactivity with proinsulin. Fasting plasma glucose (FPG), total serum cholesterol, triglycerides, and high-density lipoprotein (HDL) cholesterol levels were measured enzymatically on a Beckman AU 5800 analyser (Beckman-Coulter, High Wycombe, UK). LDL cholesterol was calculated using the Friedewald equation. Plasma whole blood samples were analysed for HbA1c on a Menarini Diagnostics HB9210 premier (A. Menarini Diagnostics Ltd., Winnersh, Wokingham, UK) [16]”.

### 2.4. SOMA-Scan Assay

Slow Off-rate Modified Aptamer (SOMA)-scan plasma protein measurement [16] was used to determine a panel of proteins reported to predictive and/or diagnostic for development of T2D (Table S1). “The SOMAscan assay used to quantify proteins was performed on an in-house Tecan Freedom EVO liquid handling system (Tecan Group, Maennedorf, Switzerland) utilizing buffers and SOMAmers from the SOMAscan HTS Assay 1.3 K plasma kit (SomaLogic, Boulder, CO) according to manufacturer’s instructions and as described previously [16,17,18]. The assay was performed in 96-well plates containing up to 85 plasma samples, 3 quality control and 5 calibrator plasma samples. Briefly, EDTA plasma samples were diluted into bins of 40%, 1% and 0.05% and incubated with streptavidin-coated beads immobilized with dilution-specific SOMAmers via a photocleavable linker and biotin. After washing, bound proteins were first biotinylated and then released from beads by photocleaving the SOMAmer-bead linker. The released SOMAmer-protein complex was treated with a polyanionic competitor to disrupt non-specific interactions and recaptured on the second set of streptavidin-coated beads. Thorough washing was performed before 5′ Cy3 fluorophore labelled SOMAmers were released under denaturing conditions, hybridized on microarray chips with SOMAmer-complementary sequences, and scanned using the SureScan G2565 Microarray Scanner (Agilent, Santa Clara, CA, USA). Initial Relative Fluorescent Units (RFUs) were obtained from microarray intensity images using the Agilent Feature Extraction Software (Agilent, Santa Clara, CA, USA). Raw RFUs were normalized and calibrated using the software pipeline provided by SomaLogic”.

### 2.5. Data Processing and Analyses

“Initial Relative Fluorescent Units (RFUs) were obtained from microarray intensity images using the Agilent Feature Extraction Software (Agilent, Santa Clara, CA, USA), normalized and calibrated using the SomaLogic software pipeline. Samples with a high degree of hemolysis (Haptoglobin log RFU < 10) were excluded from the analysis. Statistical analyses were performed on log_2_ RFU values using R version 3.5.2 (R Foundation for Statistical Computing, Vienna, Austria) including base R package. Data handling and differential protein expression were analyzed using the autonomics and limma [19] packages. For differential protein analysis, we applied limma models containing contrasts between timepoints, as well as contrasts between healthy and patients with diabetes at single timepoints. In both models, blocking by patient ID was performed to account for random effects. Batch effect correction was performed by adding batch as a covariate to the model. Limma obtained *p* values were corrected using the Benjamini-Hochberg method” [20].

### 2.6. Statistical Analysis

Changes in proteins diagnostic or predictive of β-cell dysfunction in response to a hyperinsulinemic clamp intervention have not been detailed in any previous study on which to base a power calculation. “Sample size for pilot studies has been reviewed by Birkett and Day [21] where they concluded that a minimum of 20 degrees-of-freedom was required to estimate effect size and variability. Hence, we needed to analyze samples from a minimum of 20 patients per group. Data trends were visually evaluated for each parameter and non-parametric tests were applied on data that violated the assumptions of normality when tested using the Kolmogorov-Smirnov Test. Comparison between groups was performed at each timepoint using Student’s *t*-test. A *p*-value of <0.05 was considered statistically significant. The sample size was too small to adjust for baseline covariates. Statistical analysis was performed using Graphpad Prism (San Diego, CA, USA)” [22].

### 2.7. Protein-Protein Interaction; STRING Analysis

Protein–Protein Interactions for the proteins differentially expressed in plasma of T2D versus control subjects were visualized using STRING 11.0 (Search Tool for the Retrieval of Interacting Genes) (https://string-db.org/) (accessed on 2 December 2021).

## 3. Results

### 3.1. Baseline Differences between T2D and Control in Levels of Plasma Proteins Reported to Predict β-Cell Function

At baseline, of the 43 plasma proteins measured by SOMA-scan as purported to be diagnostic and/or prognostic for T2D, 9 proteins were altered in this T2D cohort.

**Figure 1:**
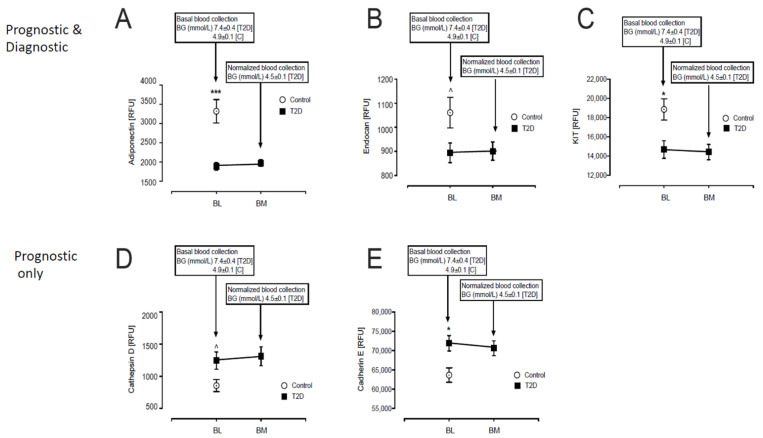
Comparison of the prognostic and diagnostic proteins of β-cell dysfunction in plasma at BL and post-iatrogenic induction of euglycemia (BM) in T2D subjects. Blood sampling for proteomics was performed at baseline (BL) in controls (white circles) and T2D (black squares) and again at euglycemia (BM) for the T2D subjects. Proteomic (Somalogic) analysis of prognostic and diagnostic proteins of β-cell dysfunction was undertaken for Adiponectin (**A**), Endocan (**B**), Mast/stem cell growth factor receptor-Kit (KIT) (**C**), Cathepsin-D (**D**), Cadherin-E (**E**). ^ *p* < 0.05; * *p* < 0.01; *** *p* < 0.0001. BG, blood glucose; RFU, relative fluorescent units; T2D, type 2 diabetes.

Of the 13 prognostic and diagnostic proteins, three were lower in T2D: Adiponectin (1885 ± 100 vs. 3319 ± 304 RFU, T2D vs. control, *p* < 0.0001), Endocan (894 ± 41 vs. 1061 ± 64 RFU, T2D vs. control, *p* < 0.05) and Mast/stem cell growth factor receptor-Kit (KIT) (14,690 ± 905 vs. 18,863 ± 1104 RFU, T2D vs. control, *p* < 0.01) (Figure 1A–C).

Of the 14 prognostic only proteins, the levels of two were higher in T2D: Cathepsin-D (1247 ± 133 vs. 857 ± 93 RFU, T2D vs. control, *p* < 0.05) and Cadherin-E (71,880 ± 2007 vs. 63,688 ± 1882 RFU, T2D vs. control, *p* < 0.005) (Figure 1D–E).

**Figure 2:**
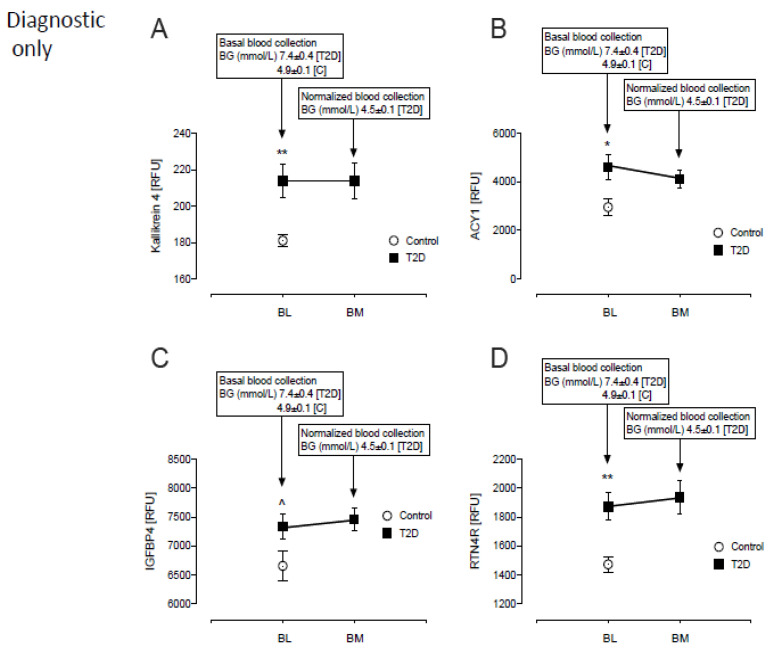
Comparison of only the diagnostic proteins of β-cell dysfunction in plasma at BL and post-iatrogenic induction of euglycemia (BM) for the T2D subjects. Blood sampling for proteomics was performed at baseline (BL) in controls (white circles) and T2D (black squares) and again at euglycemia (BM) in T2D subjects only. Proteomic (Somalogic) analysis of diagnostic proteins of β-cell dysfunction was undertaken for Kallikrein-4 (KLK4) (**A**), Aminoacylase-1 (ACY1) (**B**), Insulin-like growth factor-binding protein-4 (IGFBP4) (**C**) and Reticulon-4 receptor (RTN4R) (**D**). ^ *p* < 0.05; * *p* < 0.01; ** *p* < 0.001. BG, blood glucose; RFU, relative fluorescent units; T2D, type 2 diabetes.

Of the 16 diagnostic only proteins, 4 were higher in T2D: Kallikrein-4 (214 ± 9 vs. 181 ± 3, T2D vs. control, *p* = 0.001), Aminoacylase-1 (4616 ± 514 vs. 2956 ± 346 RFU, T2D vs. control, *p* = 0.001), Insulin-like growth factor-binding protein-4 (IGFBP4) (7334 ± 212 vs. 6658 ± 255 RFU, T2D vs. control, *p* < 0.05) and Reticulon-4 receptor (RTN4R) (1872 ± 93 vs. 1474 ± 53, T2D vs. control, *p* < 0.001) (Figure 2A–D).

Protein levels were unchanged from baseline in response to glucose normalization in T2D (Figure 1 and Figure 2).

### 3.2. Baseline Correlations of Age, Body Mass Index, Homeostatic Model Assessment for Insulin Resistance and Homeostatic Model Assessment of β-Cell Function with Levels of Plasma Proteins Reported to Predict β-Cell Function

**Figure 3:**
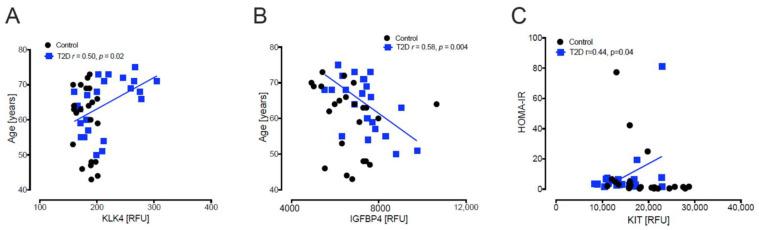
Correlation analyses of proteins. For age, there was a positive correlation with Kallikrein-4 (KLK4) (**A**) and a negative correlation with Insulin-like growth factor-binding protein-4 (IGFBP4) (**B**) in T2D (blue squares). No correlations were observed with age in controls (black circle). For HOMA-IR, there was a positive correlation with Mast/stem cell growth factor receptor-Kit (KIT) (**C**) in T2D.

Baseline levels of the 9 differentially expressed plasma proteins were correlated with age, body mass index (BMI), Homeostatic Model Assessment for Insulin Resistance (HOMA-IR) and Homeostatic Model Assessment of β-cell Function (HOMA-B). Significant correlations are detailed below and shown in Figure 3.

KLK4 (r = 0.50, *p* = 0.02) (Figure 3A) correlated positively with age in T2D only, whilst IGFBP4 (r = 0.58, *p* = 0.004) (Figure 3B) correlated negatively with age in T2D only.

In T2D, KIT (r = 0.44, *p* = 0.04) correlated positively with HOMA-IR (Figure 3C). No significant correlations of the proteins with HOMA-B were found.

**Figure 4:**
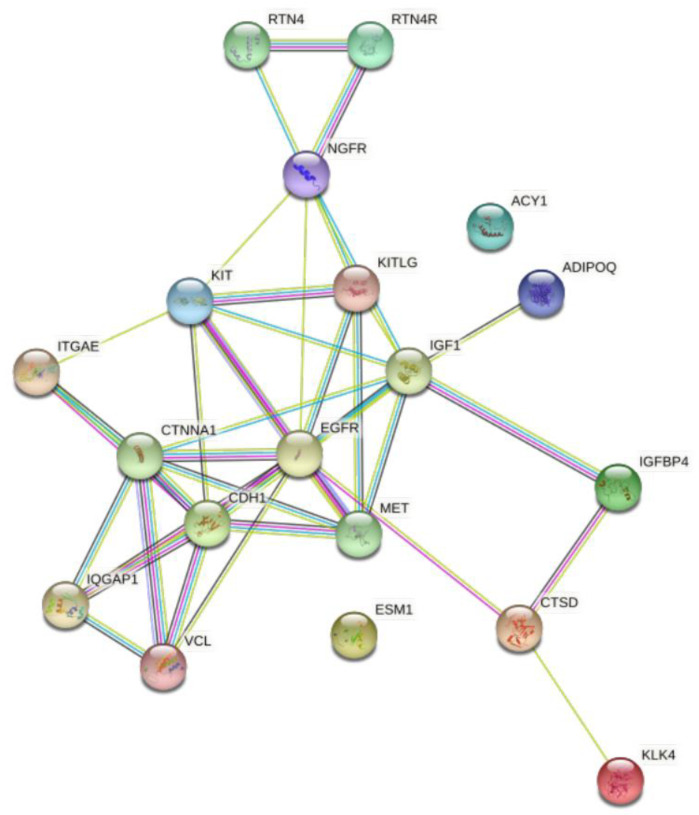
STRING interaction network showing the interactions of the prognostic and diagnostic proteins of β-cell dysfunction in plasma. STRING 11.0 was used to visualize the known and predicted protein-protein interactions for proteins identified by SOMAscan assay in plasma of T2D vs. control subjects (https://string-db.org/) (accessed on 2 December 2021).

STRING analysis illustrates the relationship between the differentially expressed proteins between the T2D and control groups (Figure 4).

## 4. Discussion

In this study, we report that there were differences in 9 of the 43-proteins that were suggested to be diagnostic or prognostic biomarkers for T2D [15]. These included Adiponectin, Endocan and KIT, which were lower in T2D, whilst other markers—namely, Cathepsin-D, Cadherin-E, Kallikrein-4, Aminoacylase-1, Insulin-like growth factor-binding protein-4 (IGFBP4) and Reticulon-4 receptor (RTN4R)—were higher in T2D. This suggests that, with the onset of T2D, there is a decrease in the discrimination of these biomarker proteins, with 34 proteins no longer being discriminatory; however, 9 proteins remained different to controls that may be of importance in terms of clinical application. Of those proteins that differed to controls, KLK4 positively correlated and IGFBP4 negatively correlated with age in T2D, suggesting that these proteins may require an age-related reference range. Interestingly, only one of the 9 proteins showed any correlation with HOMA, KIT positively correlating with HOMA-IR in T2D subjects, and none of the proteins correlated with HOMA-B, though this is not perhaps surprising, as it is well recognized that HOMA is a poor marker of β-cell mass and function [23].

For two of the three proteins that were decreased in T2D compared to controls, our findings here are in accord with the findings reported in IGT, where adiponectin and endocan were two of the top differentially expressed proteins that were found to be decreased in those with IGT and reduced β-cell sensitivity [15]. Low adiponectin levels have previously been linked to insulin resistance and β-cell function [24,25,26]; adiponectin levels are inversely correlated with body fat and have been purported to be a biomarker of adipose tissue health [4,27,28]. Endocan is a proteoglycan expressed in endothelial cells and adipocytes and low levels are associated with inflammation and visceral obesity [29,30]. Mast/stem cell growth factor receptor-Kit (KIT) was not identified as differentially expressed in the IGT cohort [15], and therefore is a novel biomarker reported here. KIT is a tyrosine protein kinase that serves as a cytokine cell surface receptor; following binding, it activates several key signaling pathways, such as AKT1 through phosphorylation of PIK3R1, the MAP kinases and STAT family members, and is expressed in mature β-cells [31].

For six of the nine proteins that were increased in T2D compared to controls, our findings are in accord with the findings reported in IGT [15]. Cathepsin-D has been reported to be reduced in the islets of T2D [32], though patients with newly diagnosed type 2 diabetes demonstrated significantly higher circulating cathepsin D concentrations than controls [33], perhaps reflecting its having been reported as a marker of β-cell dysfunction as well as insulin resistance [34]. E-Cadherin has been reported to have an important role in the control of β-cell mass in a rodent molecular knockdown model [35] and may be related to β-cell mass, survival [36,37] and function in human [38]. Kallikrein-4 is found in the pancreas but, pertinent to its role here, increased levels have been found to decrease E-Cadherin in some cell models [39] and it therefore may modulate the beneficial effect of E-Cadherin on β-cell mass. IGFBP4 is one of the so-called disallowed genes, that is repressed to prevent β-cell proliferation, and is reported to be higher in alpha cells than β-cells in the pancreas [40,41]. RTN4R is necessary for intracellular calcium entry and endoplasmic reticulum function [42], and is an essential regulator of glucose-stimulated insulin release in the pancreas [43]. No specific function or relationship to β-cell mass has been reported for Aminoacylase-1. Therefore, taken together, it can be seen that these proteins that are elevated in T2D appear to have role synergies related to the β-cell.

In response to glucose normalization in T2D, there were no changes in the levels of the 9 differentially expressed proteins, suggesting that their levels are independent of glucose variability. This also suggests that the changes were not due to an acute phase response that may be seen for other proteins following glucose normalization in this model [22].

Algorithms of therapeutic intervention in T2D, usually initiating with metformin therapy, are included in a number of published guidelines, such as that of the American Diabetes Association [14]. When glycemic control is not optimized, then additional medications are added sequentially with assessment every three to six months. In the presence of relative or absolute endogenous insulin loss, insulin therapy is required [14]. Often insulin therapy is the last therapy to be implemented, and there are considerable barriers for its initiation [44]; additionally, it is well recognized that necessary insulin therapy may be delayed significantly due to a number of factors [45]. The clinical therapeutic paradigm is based on clinical treatment failure measured by a response in the glycated hemoglobin or fasting blood glucose. This approach is in part due to a lack of readily available laboratory measurements that may guide therapy in routine practice. Biomarkers, such as those reported here, may complement clinical decision making towards initiation of exogenous insulin therapy if the biomarkers identify and quantify critically compromised β-cell mass/function.

In Somascan technology, DNA aptamers, named SOMAmers (slow off-rate modified aptamers), are constructed for each protein and quantified using DNA microarray technology. SOMAmers have stable chemical structures and recognize proteins with high binding affinities. The SOMAmer mixture quantitatively reflects the original protein concentration. This technology has the advantage of high sensitivity and specificity with median intraplate CV in the ~3–4% range [46]. However, like most proteomic discovery platforms, it is more suited for population studies than individual patient studies, as many reference ranges for the proteins have not been established. Whilst the technology has been used for other purposes in diabetes complications [22,47,48,49], it has scarce use to track T2D’s chronicity as the major issues are its availability and affordability, as the technology is very expensive and, therefore, there is limited accessibility to the platform.

The strengths of this study include the use of the state-of-the-art Somascan aptamer proteomic platform that was similarly utilized for these biomarkers previously [15], that the T2D population had a relatively short diabetes duration and that the populations were matched for age, though not BMI. The limitations of this study include relatively small numbers, and that a longer diabetes disease duration with proteomic measurement would be important to define if indeed the purported biomarkers remain robust over time. Also, it is unknown if the results reported here in this Caucasian population would hold for differing ethnic populations. Fundamental studies relating the expression of these proteins to β-cell mass/function need to be undertaken to determine if the biomarkers reflect quantitative changes. Future confirmatory studies in T2D, with a larger population encompassing a wider duration of diabetes, would then be needed.

In conclusion, these data suggest that these 9 proteins that are unaffected by glucose variability may constitute a focused biomarker panel, useful for assessing β-cell dysfunction in T2D that may facilitate future clinical decision-making on insulin therapy initiation.

## Figures and Tables

**Table 1:** Demographic and clinical characteristics of the study participants. Data are presented as mean ± SD.

Baseline	Type 2 Diabetes (n = 23)	Controls (n = 23)	*p*-Value
Age (years)	64 ± 8 (66)	60 ± 10 (63)	0.15
Sex (M/F)	12/11	11/12	0.77
BMI (kg/m^2^)	32 ± 4 (32)	28 ± 3 (27)	0.001
Duration of diabetes (years)	4.5 ± 2.2 (5.0)	N/A	
HbA1c (mmol/mol)	51.2 ± 11.4 (50.0)	37.2 ± 2.2 (37.0)	<0.0001
HbA1c (%)	6.8 ± 1.0 (6.7)	5.6 ± 0.2 (5.5)	<0.0001
Fasting plasma glucose (mmol/L)	7.6 ± 0.4	4.9 ± 0.1	<0.0001
Total cholesterol (mmol/L)	4.2 ± 1.0 (4.1)	4.8 ± 0.67 (4.9)	0.02
Triglyceride (mmol/L)	1.7 ± 0.7 (1.5)	1.34 ± 0.6 (1.3)	0.06
HDL-cholesterol (mmol/L)	1.1 ± 0.3 (1.1)	1.5 ± 0.4 (1.4)	0.002
LDL-cholesterol (mmol/L)	2.27 ± 0.8 (2.1)	2.7 ± 0.7 (2.8)	0.06
CRP (mg/L)	3.0 ± 2.7 (1.9)	5.1 ± 10.3 (2.1)	0.33
Insulin (IU/mL)	13.7 ± 7.6	21.6 ± 44.2	0.41
HOMA-IR	4.8 ± 3.8	4.8 ± 9.6	0.99
HOMA-B	173 ± 467	384 ± 762	0.26

BMI: Body mass index, HDL-cholesterol: High density lipoprotein cholesterol, LDL-cholesterol: Low density lipoprotein cholesterol, CRP: C-reactive protein. HbA1c: Hemoglobin A1c; HOMA-IR: Homeostatic Model Assessment-Insulin Resistance; HOMA-B: Homeostatic Model Assessment-β-cell function.

## Data Availability

All the data for this study will be made available upon reasonable request to the corresponding author. Because of the participant consent obtained as part of the recruitment process, it is not possible to make these data publicly available.

## Supplementary Materials

The following are available online at https://www.mdpi.com/article/10.3390/metabo12030196/s1, Table S1: The 43 proteins previously reported as prognostic and/or diagnostic for development of type 2 diabetes (T2D) that were measured in the T2D and control cohorts in this study.
