# Supplementary material for: Diagnostic and Prognostic Protein Biomarkers of β-Cell Function in Type 2 Diabetes and Their Modulation with Glucose Normalization
Source: Metabolites. 2022 Feb 22;12(3):196. doi: 10.3390/metabo12030196 (PMC8950787; doi:10.3390/metabo12030196)
Supplement: Supplementary file 1 [file metabolites-12-00196-s001.zip › metabolites-1580577-supplementary.pdf]

Supplementary Table S1. The 43 proteins previously reported as prognostic and/or diagnostic for development of type 2 diabetes (T2D) that were measured in the T2D and control cohorts in this study.

	UniProt	EntrezGeneSymbol	Target	Preferred Name
**Prognostic & Diagnostic**	Q15848	ADIPOQ	Adiponectin	ADIPOQ
	Q9NQ30	ESM1	Endocan	ESM1
	Q15828	CST6	Cystatin M	CST6
	P04196	HRG	HRG	HRG
	P01009	SERPINA1	a1-Antitrypsin	SERPINA1
	P12318	FCGR2A	FCG2A	FCGR2A
	P31994	FCGR2B	FCG2B	FCGR2B
	P10721	KIT	SCF sR	KIT
	P03951	F11	Coagulation Factor XI	F11
	Q6UX15	LAYN	Layilin	LAYN
	Q9BU40	CHRDL1	CRDL1	CHRDL1
	Q9BZZ2	SIGLEC1	Sialoadhesin	SIGLEC1
	O00533	CHL1	CHL1	CHL1
**Prognostic only**	P07339	CTSD	Cathepsin D	CTSD
	O60911	CTSV	Cathepsin V	CTSV
	P32004	L1CAM	NCAM-L1	L1CAM
	P00746	CFD	Factor D	CFD
	Q00535, Q15078	CDK5 CDK5R1	CDK5/p35	CDK5
	P02671, P02675 P02679	FGA FGB FGG	Fibrinogen	FGA
	P22748	CA4	Carbonic Anhydrase IV	CA4

	Q9NS85	CA10	Carbonic Anhydrase X	CA10
	Q13253	NOG	Noggin	NOG
	Q9NR61	DLL4	DLL4	DLL4
	P05231	IL6	IL-6	IL6
	Q9UK05	GDF2	GDF2	GDF2
	P12830	CDH1	Cadherin E	CDH1
	P01584	IL1B	IL-1b	IL1B
**Diagnostic only**	Q9Y337	KLK5	kallikrein 5	KLK5
	Q9Y5K2	KLK4	Kallikrein 4	KLK4
	Q99062	CSF3R	G-CSF-R	CSF3R
	P55289	CDH12	Cadherin-12	CDH12
	P08833	IGFBP1	IGFBP-1	IGFBP1
	Q03154	ACY1	Aminoacylase-1	ACY1
	Q9Y264	ANGPT4	Angiopoietin-4	ANGPT4
	P13725	OSM	OSM	OSM
	P22692	IGFBP4	IGFBP-4	IGFBP4
	P23280	CA6	Carbonic anhydrase 6	CA6
	P28908	TNFRSF8	CD30	TNFRSF8
	O75015	FCGR3B	FCG3B	FCGR3B
	Q9BZR6	RTN4R	Nogo Receptor	RTN4R
	P48357	LEPR	sLeptin R	LEPR
	P02751	FN1	Fibronectin	FN1
	P20333	TNFRSF1B	TNF sR-II	TNFRSF1B
